# Do we need postoperative antibiotics in supratentorial clean neurosurgery? A prospective, randomized, single-blind, and placebo-controlled study

**DOI:** 10.55730/1300-0144.5506

**Published:** 2022-06-18

**Authors:** Taha Şükrü KORKMAZ, Tufan Agah KARTUM, Gülçin BAŞ, Eren Fatma AKÇIL, Rahşan KEMERDERE, Seher Naz YENİ, Taner TANRIVERDİ

**Affiliations:** 1 Department of Neurosurgery, Cerrahpaşa Medical Faculty, İstanbul University-Cerrahpaşa, İstanbul, Turkey; 2 Department of Anesthesiology and Intensive Care Cerrahpaşa Medical Faculty, İstanbul University-Cerrahpaşa, İstanbul, Turkey; 3 Department of Neurology, Cerrahpaşa Medical Faculty, İstanbul University-Cerrahpaşa, İstanbul, Turkey

**Keywords:** Antibiotic prophylaxis, clean neurosurgery, postoperative infections

## Abstract

**Background/aim:**

To assess the efficacy of postoperative antibiotics on postoperative infection in clean supratentorial craniotomies.

**Material and methods:**

This study is a prospective, randomized, single-blind, and placebo-controlled clinical trial that included consecutive patients who underwent clean supratentorial craniotomy between November 2017 and September 2020 and evaluated the effectiveness of postoperative antibiotic prophylaxis on postoperative infection.

**Results:**

A total of 80 patients were included and the whole group was divided into two groups. Group A included patients who received antibiotic prophylaxis and group B who did not receive antibiotic prophylaxis after surgery. Each group included the same number of patients (40 patients in each). Two patients showed postoperative infection, and both were in group B. No significant difference was found regarding postoperative infection between the two groups (p = 0.15). The rate of postoperative infection was found to be 2.5% in the whole group (2 cases out of 80) and it was 5% in group B (2 cases out of 40).

**Conclusions:**

Our results showed that antibiotic prophylaxis after a clean supratentorial craniotomy has no effect on the prevention of postoperative infection and we do not suggest using antibiotic prophylaxis after clean supratentorial neurosurgery.

## 1. Introduction

Postoperative infections (PI) are one of the most important and challenging clinical problems for neurosurgeons because when it is inappropriately managed, it can lead to high morbidity and mortality rates. The reported incidence of PI rates in neurosurgery are 0.8% and 8%, respectively [1–5]. The rate in clean craniotomy is even lower, account for almost 1% and gram-positive Staphylococci have been reported to be the most common causative pathogens [3]. Apart from threatening life of patients, PI lead also to longer hospital stay and increase cost. In neurosurgery, PI generally include wound and bone flap infections (osteomyelitis), meningitis, encephalitis and abscess. Neurosurgeons always keep themselves alert when they make daily visits after surgery related to wound infection because if not managed properly, it can lead to serious complications such as meningitis, abscess and even death.

After Malis et al. [6] reported no infection in a high number of major clean neurosurgery with antibiotic prophylaxis (AP) in 1979, a series of randomized-controlled trials (RCTs) [7–11] appeared in the literature that supported the use of AP in neurosurgery. Furthermore, some meta-analysis [7,12] and retrospective studies [13] concluded that AP was effective in preventing PIs. However, widespread use of AP resulted in drug abuse, drug-resistance, changes in bacterial spectrum and high cost. As time passes, use of AP was started to be questioned after some clinical studies were reported [13,14]. Some underlined that although AP was effective in decreasing surgical site infections (SSI) [2], it had no effect on meningitis [8,9,15] and some clinical studies reported that AP is not valid in clean neurosurgery [16]. Thus, there is no common consensus on the use of AP in clean neurosurgery and the debate still exists in this modern era.

In addition of these discussions, there is no common consensus on antibiotics administration after surgery and prospective studies in literature are inadequate. It is reported in the U.S. Food and Drug Administration (FDA) database on cefazolin antibiotics that cefazolin can be continued for 3 to 5 days in the post-op period^1^. The aim of this prospective, randomized, single-blind, placebo-controlled study is to provide data on whether clean supratentorial craniotomies need postoperative antibiotics.

## 2. Material And methods

### 2.1. Study design

This prospective and randomized clinical trial included patients who underwent elective supratentorial clean craniotomy due to supratentorial pathology including tumor and epilepsy between November 2019 and February 2021 in the Department of Neurosurgery, Cerrahpaşa Medical Faculty, İstanbul University-Cerrahpaşa, İstanbul, Turkey. Detailed informed consent was signed by all patients before surgery. All patients received prophylactic antibiotics during the surgery. In the postoperative period half of the patients were received antibiotics, the other half did not. Randomization for postoperative antibiotics were achieved with a computerized random list. Medical staff was aware of the postoperative treatment, but patients were not. All patients were operated by the same surgeon with the same surgical preparation and surgical technique. The study was approved by local Ethics Committee of our hospital. We have to state that at the beginning of the study, we planned to include as many patients as possible; however, we had to terminate the study early because of COVID-19 pandemic.

### 2.2. Treatment protocol

Every patient received 2 g of cefazolin, a first-generation cephalosporin, just following induction of anesthesia. Depending on duration of surgery, appropriate dose of antibiotic was given at every 4-h interval. Ciprofloxacin, a fluoroquinolone antibiotic, was planned for patients who had cephalosporin allergy. According to length of hospital stay, the patients in the postoperative antibiotic group (Group A) received the same antibiotic as U.S. FDA had approved as the daily therapeutic dose^2^ (three times a day; daily dose of 3 g of cefazolin) and other patients in the nonantibiotic group (Group B) received a placebo (0.9% saline in water) of identical appearance until discharge.

### 2.3. Patients

In order to provide homogeneous results and decrease the study bias, some selection criteria were applied. Only adult patients (≥18 years of age) who underwent elective supratentorial clean craniotomy were included. Any patient who had any sign of infection during preoperative measures including laboratory and radiology studies was excluded. Two patients in whom the frontal sinus were opened inadvertently during craniotomy and six patie nts who needed a device for cerebrospinal fluid drainage after surgery were excluded. One patient who underwent immediate reoperation for postoperative surgical complication was excluded. Any patient who was on chemotherapy and on long-term steroid therapy for any reason was also excluded. Finally, 80 patients for this prospective clinical study met the criteria and were included.

The collected data for each patient included age; sex; presenting symptom(s); neurological examination and radiological findings; laboratory measurements including C-reactive protein, leukocyte, lymphocyte, thrombocyte and neutrophil counts; dates of hospital admission and surgery; duration of anesthesiology and surgery; postoperative neurological examination findings; measurement of vital signs during hospital stay; duration of intensive care unit stay following craniotomy; placement and duration of subgaleal drains; pathological diagnosis; cost of antibiotic used and presence of PI and their management.

The same surgical preparation was applied to all patients in the operating room by the same surgical team. Immediately after induction of anesthesia and prior to surgery, surgical site was electrically clipped and dry-shaved with disposable razor blades. The head then was fixed with three-pinned head holder for final craniotomy position. Craniotomy field was then prepared with a polyvidone-iodine solution. Adhesive drapes were never used.

The follow-up procedures with the same surgeon and residents after surgery or during hospital stay consisted of 1) patient’s visit was performed every day; 2) clinical and/or neurological status was evaluated; 2) wound inspection was done every day; 3) vital sings including body temperature was measured every day; 4) hemogram parameters and electrolytes were obtained every other day; and 5) every patient was followed-up at 3-months interval after discharge. In this study, after hospital discharge, hemogram parameters and electrolyte measures including C-reactive protein were obtained at the time of suture removal (10^th^ day of surgery).

Postoperative central nervous system infections such as meningitis and cerebral abscess and SSIs were diagnosed according to the guidelines of the Centers for Disease Control [17].

## 3. Statistical analysis

In this study SPSS software (version 20.0, IBM, USA) was used for statistical analysis. The “Student’s t-test” was used to compare continuous variables. In analysis of categorical variables “chi-square test” was used. A p-value less than 0.05 was considered statistically significant.

## 4. Results

### 4.1. General information

The whole group consisted of 80 patients with a mean age of 40.75 ± 15.7 (ranging from 18 to 75 years old) years. In the group there were 39 males (48.8%) and 41 females (51.2%). The majority of patients (n = 34; 42.5%) had seizure as presenting symptom, followed by headache (n = 23; 28.7%) and numbness on one side of the body (n = 8; 10%). The rest showed several other symptoms including paresis, decrease level of consciousness, and dysphasia. In 8 patients, there were no presenting symptoms and the pathology was incidentally found on cranial magnetic resonance imaging (MRI). MRI showed the pathology on the right side in 42 (52.5%) and left side in 38 (47.5%) patients. Mean duration of anesthesia (from the induction to wake-up of the patients) was 154.12 ± 45.4 min (ranged from 60 to 280 min) and mean duration of surgery (from the skin incision to the last suture of the skin) was 107.15 ± 40.4 min (ranged from 25 to 220 min). A total of 18 patients (22.5%) required one-night stay in the intensive care unit (ICU) after surgery. In the whole group, histopathological diagnoses were as follows: astrocytoma in 25 (31.2%), meningioma in 19 (23.7%), temporal lobe epilepsy (hippocampal sclerosis) in 13 (16.2%), neuroepithelial tumor in 8 (10%), extra-temporal epilepsy in 7 (8.8%), metastasis in 5 (6.3%), and cavernoma in 3 (3.8%) patients. Mean hospital stay was 4.65 ± 2.7 days and PI was seen in only 2 (2.5%) patients. After discharged from the hospital, all patients were scheduled to be followed-up at 3-month interval in our out-patient clinic.

### 4.2. Group comparisons

Following completion of the trial, the data was evaluated and patients were divided into those who received antibiotics after surgery (group A: 40 patients) and those who did not receive antibiotics after surgery (group B: 40 patients) during hospital stay. Table 1 and Table 2 summarize some variables related to preoperative and postoperative periods, respectively. None of the variables including mean age, gender, lateralization of the pathology, preoperative C-reactive protein, and preoperative hemogram parameters including leukocyte, lymphocyte, thrombocyte and neutrophil counts showed significant difference between the groups (p > 0.05). As shown in Figure the highest mean values in C-reactive protein (CRP) were reached on second and third postoperative days. Mean duration of anesthesia and surgery also did not show significant differences between the groups (p > 0.05). Postoperative hemogram parameters and C-reactive protein were evaluated at the time of suture removal (almost 10^th^ day of postsurgery). None of the parameter showed significant difference between the groups although C-reactive protein and lymphocyte count were slightly higher in group B compared to group A. Following surgery, 5 patients (12.5 %) in group A and 13 patients (32.5 %) in group B required one-night ICU stay. On postoperative day 1, 6 patients (15 %) in group A and 10 patients (25 %) in group B; on postoperative day 2, 3 patients (7.5 %) in each group, and on postoperative day 3, only 1 patient (2.5 %) in group B had fever (≥38 °C). No significant differences were found (p > 0.05). Following days, no fever was found in both groups. Mean hospital stay was slightly higher in group B compared to group A but the difference did not reach a significant level (p > 0.05).

### 4.3. Postoperative infection

In this study PI was seen in only 2 patients (2.5% in the whole group) and these two patients were in group B (5 % in group B). Statistical analysis did not show significant difference regarding postoperative infection between the two groups (p = 0.15). Two patients who had postoperative infection merit further discussion. The first patient was 43-year-old male and operated on right temporal tumor. The histopathological diagnosis was grade-IV astrocytoma and postoperative period he had Wernicke dysphasia. The patient was discharged from the hospital on postoperative day 6. One day after discharge, the patient admitted to our clinic with drainage of serous-purulent fluid from incision, seizure and fever. The body temperature was 38.7 °C and radiological examination showed nothing abnormal. Infectious disease consultation was asked, drainage, urine and blood cultures were performed. The patient was evaluated as superficial incisional SSI, broad spectrum antibiotics were started. During hospital stay, vital signs were normal and the results of cultures were negative. Daily care was performed at the incision site. The patient was completed antibiotic therapy for 10 days and discharged without any sequela. The second was 23-year-old male and operated on the right parietal tumor. The histopathologic diagnosis was grade-III astrocytoma and the postoperative period was uneventful. The patient was discharged from the hospital on postoperative day 4. Eleven days after discharge, the patient was admitted to our clinic with drainage of serous fluid from the incision. The radiological examination showed no abscess or any sing of intracranial infection. Neurological examination showed no signs of meningitis. The patient was taken to the operating room where skin was opened and the bone flap was elevated to inspect the dura for any fistula. There was no leakage from the dura and samples from the wound material and serous fluid were taken for culture. Then the bone flap was placed and the skin was closed primarily. Infectious disease consultation was asked and depending on their advice, samples of blood and urine were also taken for culture. The patient was evaluated as superficial incisional SSI. The patient was put on 10-day antibiotic treatment. Postoperative period was uneventful and no abnormal vital signs including body temperature were found. All the cultures were negative and after completion of antibiotic treatment, the patient was discharge from the hospital without any sequela.

### 4.4. Cost analysis

In this study, each patient was received 2 g cefazolin during the surgery and the total cost of these antibiotics was 1500 Turkish Liras (TL) (ranged from 13 to 27 TL; by dollar rate almost 1.6 to 3.5 United States Dollars (USD)).

A total of 40 patients (group A) were given postoperative AP during hospital stay. The mean dose of antibiotic given was 8.92 ± 3.02 gm and the mean cost was 83,000 TL (ranging from 32.65 to 238 TL; by dollar rate almost 4 to 30 USD).

The two patients who had PI were followed in the ward for 10 days, one of them was reoperated because of wound dehiscence. When all costs are calculated, including the ward follow-ups of two patients, the antibiotics administered and the cost of surgery performed one patient, the total approximately 9600 TL.

We have to underline that the health-care costs in our country changes depending on changes of USD against TL. During preparation of this manuscript 1 USD equaled to 7.86 TL. Thus, in this study the costs of cefazolin antibiotics used and treatment of PI were almost 12,000 USD (94,000 TL = 12,000 USD).

## 5. Discussion

It is clear that proper diagnosis and timely management of PI is utmost importance not only after neurological surgery but also after surgical interventions in other specialties. As a neurosurgeon, we know that even after clean craniotomy, improper management of PI such as simple wound infection, may lead to serious complications such as meningitis, cerebral abscess and even death. That is why neurosurgeons should keep themselves alert after surgery during follow-up periods. Neurosurgeons have adopted to use AP in neurosurgery in order to decrease the number and severity of PIs. However, it should be kept in mind that AP does not entirely prevent PIs.

Widespread use of AP in neurosurgery has begun after a series of RCTs [7–11] and some meta-analysis [12,18] were reported in 1980s and 1990s. The majority of early studies reported that AP significantly decreases rate of PIs, especially meningitis and SSIs. In the current literature, there are serious critics related to early reports that they had limitations. Furthermore, it should be noted that there was no advanced surgical equipment; surgical and anesthesiological managements were not as advanced as today and also there were logistic problem that wards were not close to ICU and more than one patient in a single room.

As time passes, substantial progress has been made in disinfection processes, ICU and ward conditions, and surgical techniques and equipment. All these advancements made surgery safe and helped surgeons to diagnose and have proper management of PIs. Thus, recent retrospective studies [13–16] failed to show effectiveness of AP in PIs, especially meningitis and wound infections after clean craniotomies. These controversies in recent years led to questioning about the use of AP, especially after clean neurosurgery. They pointed out that in the early years, Cushing was able to keep wound infection rate less than 1% by using only soap and water [19]. Misuse of AP increases drug-resistance, changes bacterial spectrum and increases cost. It has also been reported that meningitis is not occurred during surgery, rather it is occurred after surgery due to cerebrospinal fluid leak and they underlined that AP has no effect on prevention of meningitis [2,7,15]. A recent retrospective data including high number of patients (808 patients) who underwent clean neurosurgery showed that AP had no preventive effect on PI and furthermore, AP decreased culture-positivity and increased multi-rug resistant bacteria. They concluded that careful surgical technique and postoperative care are more effective than AP itself on PIs [16]. In order to eliminate the debate whether AP is required in clean craniotomies, more RCTs are needed but recent studies related to AP in neurosurgery generally are retrospective series of patients or meta-analysis because we think that it is ethically difficult today to perform RCTs compared to the periods of 1980s and 1990s. Even among early trials, opposite or contradictory results were also present [20]. However, the common consensus from the studies reported so far is that, if necessary, AP should be given timely to reduce the microbial burden of intraoperative contamination level that cannot overwhelm the host’s defense.

In the literature, there are not enough RCTs regarding to postoperative antibiotics, which is an important issue. In our prospective RCT, we evaluated whether postoperative use of antibiotics during hospital stay is required in supratentorial clean craniotomies. Our findings related to PI are in line with almost all of the previously published studies which reported PI rate that ranged from 1% to 11% [5,18] and even lower in clean neurosurgery. Our postoperative infection rate was found to be 2.5% in the whole group (n = 80 patients) and 5% in no-AP group (n = 40). We did not find significant difference related to the occurrence of PI between those who had and had not AP during hospital stay. A total of 2 patients had PI and both were in no-postoperative antibiotics group. All bacterial cultures that we performed were negative but both patients evaluated superficial SSI and received antibiotics. C-reactive protein and lymphocyte counts were slightly higher in no-AP group compared to AP-group but the difference was not significant. This difference was evaluated as the patients who had postoperative infection were in no-AP group. For all the groups, the highest mean values in CRP were reached on second and third postoperative days. After these days, it started to decrease after the third day and reached the lowest mean value postoperative 10^th^ day. Depending on our results we propose that postoperative use of antibiotics is not required and should not be given after clean supratentorial craniotomies. We agree with some previously published reports that careful surgical techniques and postoperative care are more effective than postoperative antibiotics on PIs [16,20]. Duration of surgery should be kept short as much as possible by decreasing unnecessary manipulations. We are aware of that it is very difficult to discuss and compare our results with the previously published studies with respect to PIs. The main difficulty is due mainly to the differences among the study protocols and situation of the hospital and or department or clinic where you are performing neurosurgery. Some previous studies included supratentorial and infratentorial interventions [21], some had craniotomies including burr-holes and ventriculo-peritoneal shunts (VPS) [9], and some had spinal and cranial surgeries [22] and furthermore some studies included emergency patients [2,20]. Aforementioned situations clearly change infection rate such as insertion of VPS or ventricular drainage especially after infratentorial interventions that may increase the infection rate and all these interventions make comparisons challenging. In this study we included patients who underwent supratentorial clean craniotomies only and were careful to select patients with almost similar demographic characteristics.

We are sure that almost every neurosurgeon knows that inappropriate use of antibiotics with prolonged duration can cause drug resistance, changes bacterial spectrum and leads to high cost. Thus, the question that should be asked: Why majority of neurosurgeons in developing or even in developed countries, are still using postoperative antibiotics after clean neurosurgery? We think that there are three answers to this question: 1) surgeon’s habit or preferences; 2) lack of national guidelines regarding control of use of AP; and 3) low level or lack of adherence to the available national guidelines.

Inappropriate use of antibiotics is a global problem irrespective of development status of the states. High rate of inappropriate use of AP has already been reported from our country, Turkey [23,24]. Furthermore, a cross-national database study reported by World Health Organization (WHO) in 2014 showed that use of antibiotic is highest in Turkey among European countries [25]. We have to underline that prolonged use of AP after surgery is a common practice in neurosurgical departments or clinics including ours in Turkey although almost every neurosurgeon knows that PI rate is low in clean neurosurgery. Although there are well-established guidelines related to AP throughout the world, inadequacy of adherence is a big issue worldwide. A report from Iran showed that the rate of inadequate compliance to guidelines for timing of antibiotics use is 74.3% and duration of use is almost 5 days [26]. Poor adherence was also reported from other countries including Singapore [27] and Jordan [28] with respect to timing, selection, dose and duration of postoperative antibiotics use. A recent report from one of the developed countries, Australia, pointed out that 40.3% of postoperative antibiotic prescriptions was classified as inappropriate and 45.2% as noncompliant with Australian National Therapeutic Guidelines [29]. Even in Germany [30], USA [14], UK [31], and Japan [32], half or more than half of prescriptions of antibiotics were not in full compliance with the guidelines and the doctors failed to prescribe antibiotics which were associated with inappropriate timing, selection, and prolonged duration. In Turkey, there is no common consensus on AP use and no strict national guidelines that can control antibiotic use. In almost all neurosurgical facilities AP is being used and the duration is longer on postoperative days. Infectious disease specialists are the main authority that control antibiotic use in surgical specialties including our own department. Thus, surgeons feel free to prescribe antibiotics and they decide the duration of antibiotics after surgery because of lack of restrictions. Furthermore, studies from Turkey revealed that the majority of surgeons do not carry out use of AP guidelines [23,24]. The most common practice or habit among surgeons is prolonged use of postoperative antibiotics which is generally more than 5 days. Especially in private hospitals in Turkey, patients after clean craniotomies are discharged from hospital with oral antibiotics until the time of suture removal (personnel communication with colleagues worked in private hospitals).

Apart from medical consequences of inappropriate use of AP, increased cost should be taken into consideration. Cost effectiveness is vital for every country, especially for underdeveloped and developing countries because of limited sources. In accordance with the main purpose of this study, we would like to draw attention to the cost of antibiotics used unnecessarily after surgery. In our study, 40 patients used AP in the postoperative days until discharge from our hospital and the cost was 83,000 TL which almost equals to 10,600 USD. This amount may not be considered as a high price for a developed country, but it matters in under-developed and/or developing countries, like our country, Turkey, when we think about the minimum salary of a worker in Turkey in 2020 is 2324 TL which equals to almost 303 USD per month. In short, we have to think about socio-economic burden of inappropriate use of AP in the countries, such as in Turkey where the National Insurance of Health is covering more than 90% of the population throughout the country.

## 6. Limitations

The authors of this prospective study are aware of the limitations. We think that the single most important limitation of this study is the number of the patients. At the beginning of the study, we planned to include more patients but unfortunately, we had to terminate the study early due mainly to COVID-19 pandemics. Future studies should be prospective and should include a greater number of neurosurgical patients (ideally should be national or international multi-centric) in order to provide more reliable data related to use of postoperative antibiotics in clean neurosurgery.

## 7. Conclusion

Our data showed that postoperative antibiotics have no effect on PI after clean supratentorial craniotomies and no difference was found between the groups with and without postoperative antibiotic. Careful handling of surgery and postoperative care seem to be more important and effective than postoperative antibiotics.

## Figures and Tables

**Figure:**
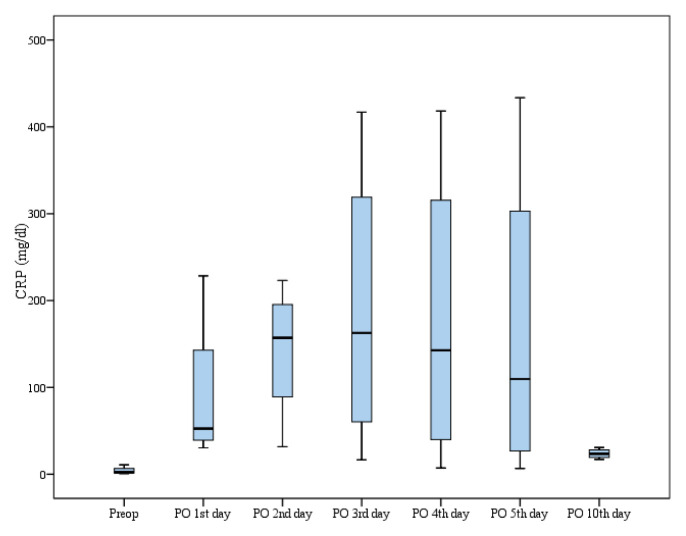
Boxplot graph of preoperative and postoperative CRP values. The highest mean values in CRP were reached on the second and third postoperative days. It started to decrease after the third day and reached the lowest mean value postoperative 10^th^ day. CRP: C-Reactive Protein (mg/dL). Preop: Preoperative. PO: Postoperative.

**Table 1:** Demographic and preoperative clinical and laboratory variables in both groups.

Variables	Group A (n = 40)	Group B (n = 40)	p-values
Mean age (years)	39.4 ± 15.2	42.0 ± 16.3	0.4
Gender (male/female)	22/18	17/23	0.2
Lateralization (right/left)	21/19	21/19	1
C-reactive protein (mg/dL)	2.23 ± 1.89	4.42 ± 11.1	0.2
Leukocyte count (10^3^ mm^3^)	8.79 ± 4.2	8.30 ± 3.6	0.5
Lymphocyte (10^3^ mm^3^)	2.04 ± 0.6	2.06 ± 0.6	0.8
Thrombocyte (10^3^ mm^3^)	265.5 ± 81.2	267.2 ± 78.4	0.9
Neutrophil (10^3^ mm^3^)	5.96 ± 4.1	5.49 ± 3.1	0.5

Group A: Patients who received postoperative antibiotic prophylaxis during hospital stay. Group B: Patients who did not receive postoperative antibiotic prophylaxis during hospital stay. None of the p-values are less than 0.05.

**Table 2:** Surgical and postoperative clinical and laboratory variables in both groups.

Variables	Group A (n = 40)	Group B (n = 40)	p-values
Mean anesthesia duration (min)	156.2 ± 51.9	152.0 ± 38.2	0.6
Mean surgery duration (min)	107.5 ± 46.0	106.7 ± 34.4	0.9
*C-reactive protein (mg/dL)	10.5 ± 8.2	11.2 ± 21.9	0.8
*Leukocyte count (10^3^ mm^3^)	10.57 ± 4.9	9.8 ± 3.5	0.4
*Lymphocyte (10^3^ mm^3^)	2.26 ± 0.6	2.42 ± 1.1	0.4
*Thrombocyte (10^3^ mm^3^)	329.1 ± 89.8	324.9 ± 75.2	0.8
*Neutrophil (10^3^ mm^3^)	7.44 ± 4.5	6.69 ± 3.0	0.3
Mean hospital stay (day)	5.25 ± 2.3	6.05 ± 3.0	0.1
Postop. infection(s)	0	2	0.15

Group A: Patients who received postoperative antibiotic prophylaxis during hospital stay. Group B: Patients who did not receive postoperative antibiotic prophylaxis during hospital stay. None of the p-values are less than 0.05. Postop: Postoperative.

* Hemogram parameters at the 10^th^ day of surgery (time of suture removal).

## Footnotes

1 U.S. Food and Drug Administration (2011). Cefazolin for Injection, USP. [online]. Website https://www.accessdata.fda.gov/drugsatfda_docs/label/2013/065247s011lbl.pdf [accessed 15 March 2022]

2 U.S. Food and Drug Administration (2011). Cefazolin for Injection, USP. [online]. Website https://www.accessdata.fda.gov/drugsatfda_docs/label/2013/065247s011lbl.pdf [accessed 15 March 2022]
